# An Illustrated Encyclopedic Medical Dictionary

**Published:** 1891-03

**Authors:** 


AN ILLUSTRATED ENCYCLOPEDIC MEDICAL DICTIONARY. Being a dictionary of the technical terms used by writers on medicine and the collateral sciences, in the Latin, English, French and German languages. By FRANK P. FOSTER, M. D., editor of the *New York Medical Journal*, assisted by eleven collaborators. Vol. II. Cac – Fasay. With illustrations. Quarto, pp. 792. New York : D. Appleton & Co. 1890.

When the first volume of this comprehensive dictionary appeared, we expressed our approbation of the work (see JOURNAL, August, 1888,) in measured and considerate terms. After a careful examination of the present volume, we see no reason to modify our opinion, but rather to intensify the judgment then advanced. The elaborateness of the plan, the precision of its details, and the accuracy of execution at once command our attention and admiration.

In turning its pages with reference to definitions, one is particularly impressed with the clearness of expression on that point without diffuseness. Here we find thousands of strange words that betray with startling abruptness our ignorance of medical terms, or else delight us with new-found old friends that had been forgotten because of "innocuous desuetude."

One of the strong features of this dictionary is the excellent character of the illustrations, which everywhere betray artistic merit and truthful accuracy. It is a pity that we have to wait so long for the successive volumes to appear, but this could not well be remedied in a work on such a grand scale. Perhaps something may be thought, if not said, in criticism on this score, but we prefer to possess our souls in patience, when such a rich storehouse of knowledge is being opened to us, rather than to carp at the tardiness of the person who holds the key. Dr. Foster and his able staff deserve nothing but praise for the work in which they are engaged, and, when completed, it may well take rank as the foremost literary achievement of the century in the field of medicine. The publishers are also entitled to the highest commendation for their enterprise and the perfection of their work.

